# Global-local processing relates to spatial and verbal processing: implications for sex differences in cognition

**DOI:** 10.1038/s41598-017-11013-6

**Published:** 2017-09-05

**Authors:** Belinda Pletzer, Andrea Scheuringer, Thomas Scherndl

**Affiliations:** 1 Department of Psychology, University of Salzburg, Salzburg, Austria; 2 Centre for Cognitive Neuroscience, University of Salzburg, Salzburg, Austria

## Abstract

Sex differences have been reported for a variety of cognitive tasks and related to the use of different cognitive processing styles in men and women. It was recently argued that these processing styles share some characteristics across tasks, i.e. male approaches are oriented towards holistic stimulus aspects and female approaches are oriented towards stimulus details. In that respect, sex-dependent cognitive processing styles share similarities with attentional global-local processing. A direct relationship between cognitive processing and global-local processing has however not been previously established. In the present study, 49 men and 44 women completed a Navon paradigm and a Kimchi Palmer task as well as a navigation task and a verbal fluency task with the goal to relate the global advantage (GA) effect as a measure of global processing to holistic processing styles in both tasks. Indeed participants with larger GA effects displayed more holistic processing during spatial navigation and phonemic fluency. However, the relationship to cognitive processing styles was modulated by the specific condition of the Navon paradigm, as well as the sex of participants. Thus, different types of global-local processing play different roles for cognitive processing in men and women.

## Introduction

Sex differences have been reported for a variety of cognitive tasks, with varying degrees of consistency between different studies (for reviews see refs 1–3). Best documented are sex differences favoring men in spatial abilities, specifically in spatial navigation and mental rotation tasks, while there is still some dispute about sex differences in numerical, verbal and memory abilities^1^. Sex differences favoring women have however been documented for verbal fluency and verbal memory tasks^2^.

These sex differences have previously been related to men and women processing the presented stimulus materials in different ways, which has often been referred to as the use of different cognitive strategies. However, the term strategy can imply a component of awareness, i.e. participants deliberately choosing to process the stimulus material a certain way. As this is not necessarily the case in all of the tasks for which sex differences were described, we will use the term processing style instead. It has been demonstrated that certain processing styles are more beneficial for some task than others. Thus, sex differences in overall task performance may arise, if men and women differ in their use of said processing style (for a review see ref. 3). Specifically, in spatial navigation tasks, men tend to take a more allocentric perspective and use a more Euclidian approach, while women tend to take a more egocentric perspective and landmark-based approach^4–10^. Allocentric perspective-taking is characterized by a focus on cardinal directions, like north, east, south, and west, whereas egocentric perspective-taking relies on personal directions like right or left^8^. A landmark-based navigation approach relies on concrete local landmarks, whereas an Euclidian approach focuses on stable global landmarks and absolute distances^8^. It has been demonstrated that instructions requiring an allocentric perspective and Euclidian approach lead to better performance in men, while instructions requiring an egocentric perspective and landmark-based approach lead to better performance in women^9^.

In mental rotation tasks, men tend to use a more holistic approach of rotating whole objects, while women tend to use a more sequential approach of separately rotating parts of the object^11, 12^. It has been demonstrated that sex differences in mental rotation tasks disappear, after de-emphasizing the spatial nature of the task in the instructions^13^. Similarly, for number comparison tasks, it has been reported that women use a more decomposed approach of comparing single digits, while men use a more holistic approach of comparing multi-digit numbers as a whole, irrespective of whether sex differences in overall performance were observed^14^.

For verbal fluency tasks, it has been demonstrated that men tend to display stronger clustering of words, i.e. produce subsequent words out of the same sub-category, while women tend to switch more strongly between sub-categories when producing subsequent words^15–17^. Switching has been related to better performance in the task^15, 18^. For an emotional memory task, it has repeatedly been demonstrated that women tend towards remembering the details of an emotional story more strongly than men, while men tend to remember the gist of that same story more strongly than women^19^.

It was recently argued that all these processing styles share the feature of being directed towards global/holistic stimulus aspects in men, but local/detail-oriented stimulus aspects in women^3^. In that respect, they all share similarities with global-local processing, i.e. the tendency to process hierarchical stimuli as a whole or by focusing on the parts that build up the stimulus. A classic paradigm to assess global-local processing is the Navon-paradigm. Navon stimuli consist of large global structures (letters or shapes), made up of small local elements of the same kind^20^. Participants are asked to detect a target either irrespective of the level (divided attention) or specifically only among the global structures or among the local elements (focused attention). As measure of global vs. local processing, a global-advantage effect can be observed, i.e. faster responses to global as opposed to local targets. The size of the global advantage effect depends on various stimulus characteristics and is also modulated by stimulus material and attention condition. Generally larger global advantage effects were observed with letter stimuli and in the focused attention condition.

Supporting the idea that sex-dependent processing styles share similarities with global-local processing, sex differences in the Navon paradigm have previously been demonstrated by some studies^21–23^, although non-significant effects have also been reported^24^. The studies finding sex differences suggest stronger global processing in men, but stronger local processing in women^21–23^. Furthermore, sex differences in the Kimchi-Palmer task, using similar stimulus material have also suggested a global preference in men, but a local preference in women^25, 26^.

However, the hypothesis that sex-dependent processing styles in complex cognitive tasks relate to basic global-local processing at the attentional level, has not been tested directly in previous studies. To address this goal, a comprehensive study was conducted on a sample of healthy men and naturally cycling women. Participants performed a series of tasks at two time-points, which were time-locked to the low hormone follicular and high-hormone luteal phase in women. These tasks include a classic Navon paradigm as implemented in ref. 23 and a Kimchi-Palmer to comprehensively address global-local processing, as well as a spatial navigation and a verbal fluency to address cognitive processing styles. Sex differences and menstrual cycle dependent changes in the Kimchi-Palmer task, as well as in the navigation and verbal fluency task were previously described^17, 26^. Specifically we reported faster responses for global choices as compared to local choices for men compared to women in the Kimchi-Palmer task, but no menstrual cycle modulation of performance in the Kimchi-Palmer task^26^. In the navigation task, we unexpectedly found neither perspective nor navigation approach [previously termed strategy^17^] to be significantly modulated by sex of participants, although overall performance was better in men compared to women. However, as expected, perspective and approach varied along the menstrual cycle with better performance for the ego-centric perspective and landmark-based approach during the high hormone luteal cycle phase compared to the low hormone follicular cycle phase^26^. In the verbal fluency task, we found overall better performance in women compared to men, that resulted from a larger number of switching between sub-categories in women compared to men^26^. Clustering did not differ between men and women and neither clustering nor switching was modulated by menstrual cycle phase.

The goal of the present study was to relate global-local processing as assessed with the Navon paradigm and the Kimchi-Palmer task to the cognitive processing styles in the navigation and verbal fluency task.

Comparably to the Kimchi-Palmer task, we expect the global advantage effect in the Navon task (i.e. faster processing of global targets as opposed to local targets) to be larger in men compared to women and during the follicular as opposed to the luteal cycle phase. Similar results were previously observed using the same task^23^. Subsequently, we expect a latent factor of global advantage to emerge from the different conditions of the Navon paradigm and the Kimchi-Palmer task, reflecting an individual’s overall tendency to process hierarchical stimuli in a global or local manner irrespective of situational influences posed by different task conditions. We hypothesize on the one hand that this global advantage relates positively to the effects of perspective and navigation approach during navigation. On the other hand, we hypothesize that the global advantage relates positively to clustering and negatively to switching in the verbal fluency task.

## Results

### Global advantage effect

#### Sex differences

GA was subjected to a linear mixed effects model with the fixed effects session, as well as the interactive effects of sex, material and attention condition (GA ~ 1|PNr + session + sex*material*attention). Non-significant interactions were backwards eliminated using the step function of the lmerTest package.

**Figure 1:**
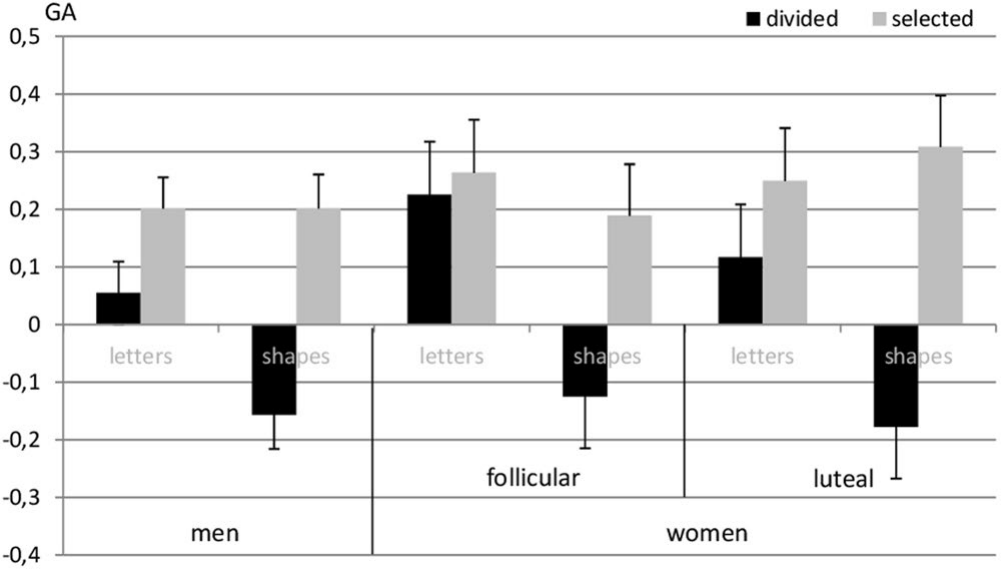
Effects of sex, material and attention condition on the global advantage effect (GA). GA was significantly larger in the selected compared to the divided attention condition and with letter stimuli compared to shape stimuli. Sex and menstrual cycle phase did not affect the GA effect.

Session had no significant effect on the GA and was therefore removed from the model. There was a significant main effect of material (*b* = −0.51, *SE*_*b*_ = 0.11, *t*_(468)_ = −4.56, *p* < 0.001) indicating a stronger GA for letter stimuli compared to shape stimuli. The significant main effect of attention condition (*b* = 0.24, *SE*_*b*_ = 0.11, *t*_(468)_ = 2.22, *p* = 0.027) indicates a stronger GA for the selected attention compared to the divided attention. The main effect of sex was non-significant and did not interact with material or attention condition. It was therefore removed from the model. There was however a significant interaction between material and attention condition (*b* = 0.51, *SE*_*b*_ = 0.16, *t*_(468)_ = 3.25, *p* = 0.001) indicating that the effect of attention condition was stronger for shape stimuli compared to letter stimuli. Results are illustrated in Fig. 1.

#### Menstrual cycle effects

In women, the main effects of material and attention, as well as the material*attention interaction were confirmed. Menstrual cycle phase had no significant effect on GA and did not interact with either material or attention (Fig. 1). It was thus removed from the model.

### Relation of GA to perspective and strategy in the 2D matrix navigation task

In the 2D matrix navigation task, better performance with an allocentric as compared to an ego-centric perspective, i.e. a positive perspective effect, as well as with an Euclidian as compared to a landmark-based strategy, i.e. a positive strategy effect, is indicative of more holistic and less detail-oriented processing. In order to be used as dependent variables, these effects, i.e. the perspective effect and effect of navigation approach [previously termed strategy effect^17^] were calculated as scaled average over the standardized mean differences for reaction times (RT) and the difference scores for error rates (ER).

In order to assess whether these effects were affected by global-local processing, they were subjected to linear mixed effects models with the fixed effects session, as well as the interactive effects of sex and GA including all 5 GA effects as separate predictors within the same model (effect ~ 1|PNr + session + sex*GA1 + sex*GA2 + sex*GA3 + sex*GA4 + sex*GA5). Non-significant interactions were backwards eliminated using the step function of the lmerTest package. Since 2 models were run for this task, p-values were Bonferroni-corrected to 0.025.

**Figure 2:**
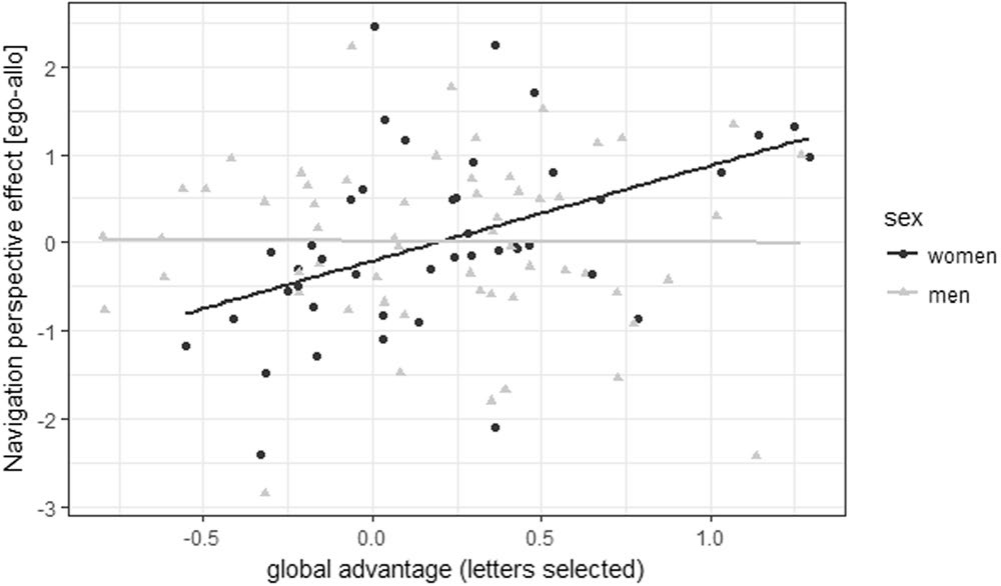
Relationship of the GA effect to the perspective effect in the 2D matrix navigation task. A larger GA effect in the letters selected condition was related to better performance with allocentric as compared to egocentric instructions in the navigation task in women, but not in men.

The perspective effect was significantly positively related to the GA effect during the letters selected condition (*b* = 0.46, *SE*_*b*_ *= *0.16, *t*_(42)_ = 2.89, *p* = 0.006), indicating the better performance with the allocentric compared to the egocentric perspective, the larger the GA effect. This relationship was furthermore modulated by a significant sex*GA interaction (*b* = −0.48, *SE*_*b*_ = 0.20, *t*_(42)_ = −2.42, *p* = 0.02), indicating that the relationship was weaker in men compared to women (Fig. 2). GA effects during the other conditions did not affect the perspective effect during navigation and were thus removed from the model.

The effect of navigation approach was not significantly related to any of the GA effects, which were thus removed from the model.

### Relation of GA to Clustering and Switching in the Verbal Fluency task

In order to assess whether clustering and switching during phonemic and semantic fluency were affected by global-local processing, average cluster size and number of switches in each task were subjected to a linear mixed effects model with the fixed effects session, as well as the interactive effects of sex and GA including all 5 GA effects as separate predictors within the same model (effect ~ 1|PNr + session + sex*GA1 + sex*GA2 + sex*GA3 + sex*GA4 + sex*GA5). Non-significant interactions were backwards eliminated using the step function of the lmerTest package. Since 4 models were run for this task, the significance-level Bonferroni-corrected to 0.0125.

**Figure 3:**
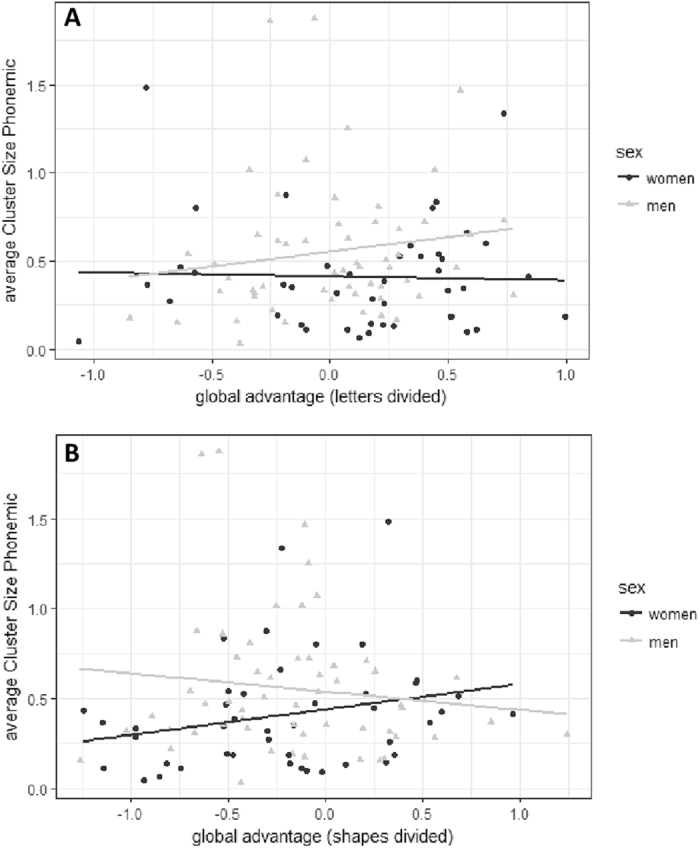
Relationship of the GA effect to clustering in the phonemic fluency task. A larger GA effect in the letters divided condition was related to larger clusters in men (**A**), while the GA effect in the shapes divided condition was related to larger clusters in women (**B**).

In the phonemic task, cluster size was significantly modulated by a significant sex*GA interaction for the letters divided condition of the Navon task (*b* = 0.49, *SE*_*b*_ = 0.17, *t*_(39)_ = 2.90, *p* = 0.007), while the main effect of GA in that condition was not significant (*b* = −0.17, *SE*_*b*_ = 0.11, *t*_(39)_ = −1.61, *p* = 0.12). This indicates a stronger positive relationship in men compared to women. Furthermore, phonemic cluster size was significantly positively related to the GA in the shapes divided condition (*b* = 0.33, *SE*_*b*_ = 0.11, *t*_(39)_ = 2.86, *p* = 0.007), i.e. participants with a larger GA produced larger Clusters. This relationship was further modulated by a significant sex*GA interaction (*b* = −0.52, *SE*_*b*_ = 0.16, *t*_(39)_ = −3.16, *p* = 0.003), indicating a stronger positive relationship in women compared to men (Fig. 3). GA during the other conditions were not related to clustering in the phonemic task and thus removed from the model. The number of switches was not significantly predicted by any of the GA effects, which were thus removed from the model. In the semantic task, no GA effect related to cluster size or the number of switches. They were thus all removed from the model.

## Discussion

The aim of the present study was to investigate whether holistic vs. detail-oriented processing styles in a variety of cognitive tasks can be related to global-local processing as assessed with two classic paradigms (the Navon task and the Kimchi-Palmer task). It was hypothesized that a general tendency towards global processing should translate into a more holistic processing style during cognitive tasks, whereas a general tendency towards local processing should translate into a more detail-oriented processing style during cognitive tasks. Accordingly it was assumed that sex differences in cognitive processing may result from sex differences in global-local processing. Indeed we found that global-local processing was related to (i) the perspective effect during spatial navigation and (ii) clustering during phonemic fluency. However, before interpreting these results in more detail it is important to point out two observations about global-local processing itself.

First, it was not possible to obtain a latent construct for global-local processing that reflects an individual’s general tendency towards global over local processing irrespective of task and stimulus condition. While it has previously been demonstrated that stimulus material (letters vs. shapes) and attention condition (divided vs. focused) influence the size of the GA effect in a Navon paradigm^23^, it was assumed that this was the result of different conditions enhancing or suppressing the general individual tendency to process hierarchical stimuli in a global or local manner. However, this does not appear to be the case. Rather, the results of the present study suggest that global-local processing is not a uniform construct in itself and that the same participant may process some stimuli in a global manner, but other stimuli in a local manner. Consequently, it has to be assumed that – if global-local processing influences cognitive processes - this diversity will translate to cognitive processing styles. This study set out to confirm that an overall tendency towards global or local processing relates to cognitive processing styles. However, the lack of such an overall tendency made it necessary to switch to an exploratory approach of determining which types of global-local processing are relevant for which cognitive tasks. Indeed, the results show that different measures of global-local processing are of different relevance for the navigation and the verbal fluency task.

Second, we did not confirm the expected sex difference in the Navon paradigm, which is contrary to previous results on the same task^23^. The fact that in the same sample, sex differences arise in one global-local task, i.e. the Kimchi-Palmer task^17^, but not in another global-local task, further supports the diverse nature of global-local processing itself. The only obvious difference between the current study and our previous study^23^, which could explain these deviating findings, is the country in which the data were collected. While age, educational level and hormonal status of participants were comparable between the two studies, the previous data set^23^ was collected on a US sample, while the current data set was collected on a middle European sample. Cultural influences on global-local processing in the Navon paradigm have repeatedly been demonstrated when comparing Asian and Caucasian samples^27, 28^. Accordingly, the question whether sex differences in global-local processing explain sex differences in cognitive processing styles also has to be re-evaluated, since not all measures of global-local processing are sexually dimorphic. Indeed our data demonstrate that some measures of global-local processing are more relevant for cognitive processing in men, while other measures are more relevant for cognitive processing in women. The Kimchi-Palmer task however, for which sex differences were previously observed, did not relate to cognitive processing styles in either the navigation or the verbal fluency task.

Following up on the first issue (the diversity of global-local processing), the following pattern of results was observed. While the global advantage during the Navon letters selected condition played a stronger role for the navigation tasks, the global advantage in the divided attention condition of the Navon task played a stronger role for the phonemic fluency task. In the divided attention condition, participants are not instructed to focus on a predefined level as in the focused attention condition. Thus, the global advantage effect in the divided attention condition reflects, how salient global or local stimuli are for a participant. The global advantage effect in the focused attention condition however, reflects how easy it is for participants to direct their attention to a pre-defined level. Thus it makes sense that the selected attention condition of the Navon task was more relevant for the task in which the cognitive processing style was pre-determined by instruction, i.e. the navigation task, while the divided attention condition of the Navon task was more relevant for the task in which the cognitive processing style was not pre-determined by instruction, i.e. the verbal fluency task.

It is furthermore interesting to note that for women, the visuo-spatial navigation task was influences by the global advantage with letter stimuli, while the verbal fluency task was influenced by the global advantage effect with shape stimuli. Thus for women, global processing of verbal stimulus material was more important for holistic processing of spatial information, while global processing of figural stimulus material was more important for holistic processing of verbal information. It has previously been suggested that the traditional global-right but local-left lateralization can be confirmed for letter stimuli, but is reversed for object stimuli, i.e. local-right but global-left^29^. This could in part explain the pattern of relationships observed in the present study. It has repeatedly been demonstrated that verbal material is preferably processed in the left hemisphere as are the global aspects of Navon shape stimuli according to ref. 29. Vice versa it has been suggested that visuo-spatial material is preferably processed in the right hemisphere, as are the global aspects of Navon letter stimuli.

Following up on the second issue (the role of global-local processing for sex differences in cognition), it is noteworthy that all relationships observed in the present study, were modulated by sex. This suggests that unlike previously assumed, sex differences in cognitive processing styles may not be the direct result of sex differences in global-local processing. Rather different types of global-local processing play a role for cognitive processing styles in men and women. Specifically, none of the global advantage effects related to holistic processing in the navigation task in men. This may however be the result of a ceiling effect in navigation performance in men, who outperformed women irrespective of perspective or strategy^17^. Furthermore, clustering during phonemic fluency related to global processing of Navon letter stimuli in men, but global processing of Navon shape stimuli in women.

In summary this study supports the idea that global-local attentional selection plays a role for cognitive processing styles. However, the lack of a uniform global-local processing construct complicates the picture. Furthermore, results of the present study suggest that sex differences in cognitive strategies do not result from sex differences in global-local processing. Rather, cognitive processing styles are differentially influenced by different aspects of global-local processing in men and women.

## Methods

### Participants

A total of 105 healthy young right-handed participants were recruited for this study, of which 5 dropped out after the first test session and for 7 inconsistencies in hormone values were observed as described in Scheuringer & Pletzer (2016, 2017). Thus 49 men (mean age: 23.67, SD = 4.13) and 44 women (mean age: 22.73, SD = 3.32) remained in the sample to be analyzed. Age of all participants ranged between 18 and 36 years and did not differ significantly between men and women (*t*_(98)_ = −1.21, *p* = 0.23). Participants were students of the University of Salzburg, who received course credits for their participation. They had all obtained general qualification for University entrance and were thus at a comparably high level of education. All women had a natural menstrual cycle of a regular duration between 21 and 36 days (mean cycle length: 29.09 days, SD = 2.53).

### Ethics statement

All participants gave their written consent to participate in the study and all methods conform to the Code of Ethics of the World Medical Association (Declaration of Helsinki).

The institutional guidelines of the University of Salzburg (Statutes of the University of Salzburg – see https://online.uni-salzburg.at/plus_online/wbMitteilungsblaetter.display?pNr=98160) state in § 163 (1) that ethical approval is necessary for research on human subjects if it affects the physical or psychological integrity, the right for privacy or other important rights or interests of the subjects or their dependents. In § 163 (2) it is stated that it is the responsibility of the PI to decide whether (1) applies to a study or not. Therefore, we did not seek ethical approval for this study. Since it was non-invasive and performed on healthy adult volunteers, who gave their informed consent to participate, (1) did not apply. Data was processed in anonymized/deidentified form. Upon arrival at the lab, participants were assigned a subject ID (v001, v002, etc.) which was used throughout the study.

### Procedure

All participants were tested twice. While men participated within an interval of around 2 weeks, women participated in two different cycle phases, i.e. the low-hormonal early follicular phase (days 1–6 of their menstrual cycle) and the high-hormonal mid-luteal phase (3–10 days after ovulation). Ovulation was calculated based on participants’ self-reports and was confirmed by commercial ovulation tests and by hormone levels analyzed in saliva after testing. During the first session, 20 women were in their follicular cycle phase and 24 women in their luteal cycle phase.

During each test-session participants completed five tasks in the following order: (i) verbal fluency task (ii) Navon paradigm, (iii) number comparison task (described elsewhere), (iv) navigation task, (v) Kimchi-Palmer task, preceded by a consent form and screening questionnaires during the first test session and followed by a quick debriefing during the second test session. All computerized tasks were presented with Presentation Software (version 0.71), 2009, Neurobehavioral Systems Inc., Albany, CA, USA).

### Navon paradigm & Kimchi-Palmer task

To obtain a comprehensive measure of global-local processing, we employed both a Navon paradigm and the Kimchi-Palmer task. We used the same Navon paradigm as described in detail in ref. 23. The Kimchi-Palmer task was described in detail in ref. 26.

#### Navon paradigm

**Figure 4:**
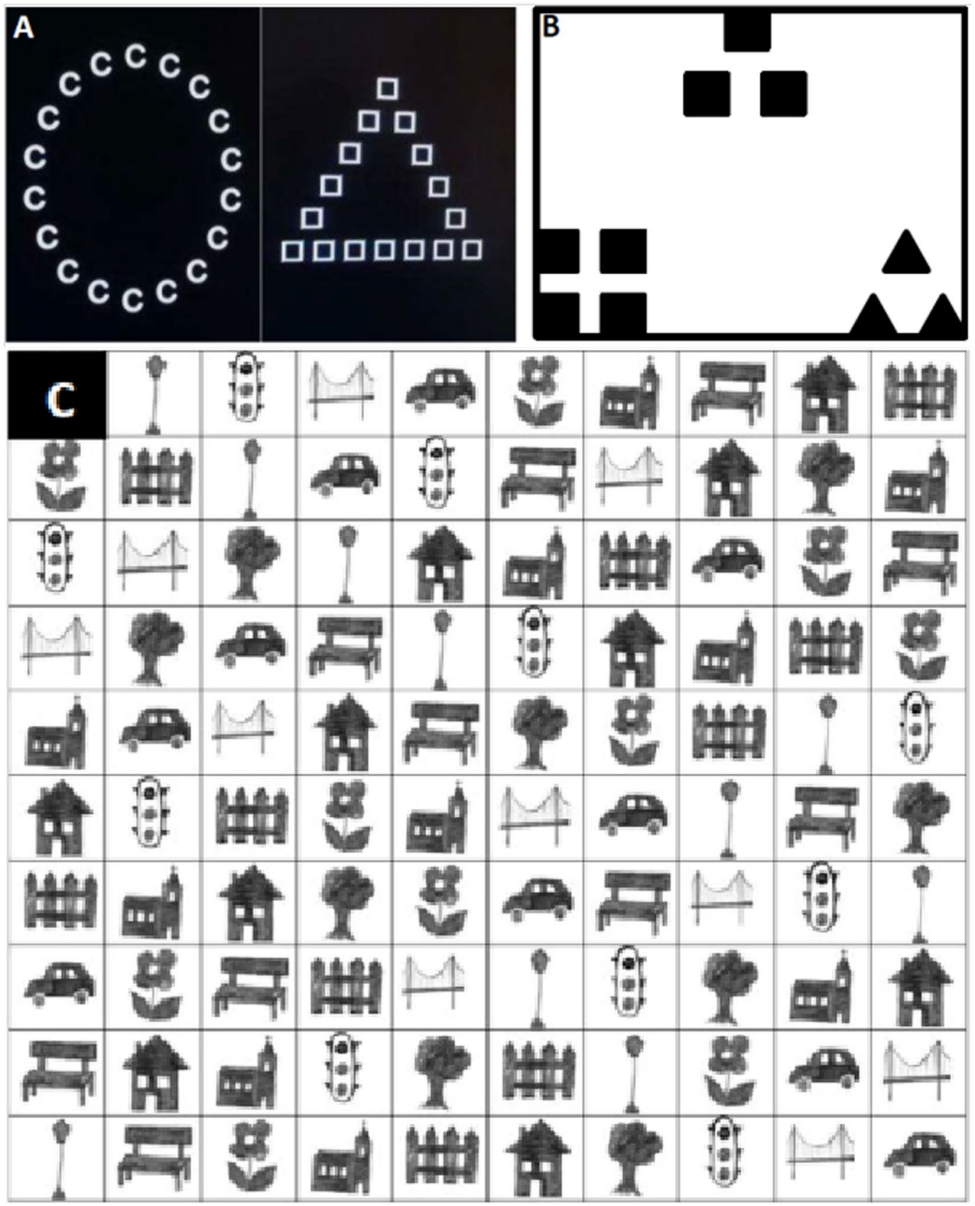
Example stimuli for (**A**) the Navon task, (**B**) the Kimchi-Palmer task and (**C**) the 2-D Matrix navigation task.

To obtain a comprehensive estimate of the global advantage effect, Navon stimuli were constructed from two materials: letters and shapes (compare Fig. 4A) and presented in two attention conditions. Both material and attention condition have previously been shown to significantly modulate the global advantage effect per se and hemispheric asymmetries in global advantage. In the divided attention condition, participants were asked to detect predefined targets at any level. In the focused attention condition, participants were asked to detect targets at only the global or local level, respectively. Reaction times over correctly solved items (RT) and error rates (ER) were recorded. As in our previous study^23^, some participants (15 men, 16 women) displayed high error rates in some Navon task categories and were thus excluded from the analyses.

#### Kimchi-Palmer task

The Kimchi-Palmer task as developed by Kimchi & Palmer^30^ was described in detail in ref. 26. Participants were presented with a triad of hierarchical figures (see Fig. 4B for an example) and had to subjectively decide as quickly as possible which of two comparison figures on the bottom matched a target figure on the top more closely. Reaction times (RT) for global and local decisions were assessed. RT more than 3 standard deviations above the mean were discarded as outliers as ref. 26.

### Cognitive tasks

Cognitive paradigms have been described in detail in ref. 17.

#### Navigation task

To evaluate spatial navigation strategies, participants completed a computerized 2D-matrix navigation task, which was adapted from an earlier paper-pencil version by ref. 9. Participant had to find their way to a target position on a 10 × 10 matrix containing 10 different symbols in each row and column (for an example, see Fig. 4C). For each item, the path to take was described in a short written instruction formulated to manipulate two possible perspectives (i.e. Allo- vs. egocentric) and two possible strategies (i.e. Euclidian vs. landmark-based) in a 2 × 2 design. Response times and accuracy were recorded for each item.

#### Verbal fluency task

To evaluate verbal abilities, as well as clustering and switching strategies, participants completed paper-pencil versions of a phonemic and a semantic fluency task in randomized order. Each condition included three different instructions. For the present analyses, only the neutral instruction presented at the beginning of the task is of relevance, as the other instructions manipulated clustering and switching. In each trial, participants were asked to write down as many words as possible within one minute, beginning with a predefined letter (phonemic) or belonging to a pre-defined category (e.g. *animals*, semantic). To assess verbal fluency strategies, the mean cluster size and the mean number of switches was counted using the rules suggested by ref. 31.

### Statistical analyses

Statistical analysis was carried out in R 3.3.2.

As a measure of global vs. local processing in the Navon task, the global advantage effect (GA) was calculated separately for each condition (letters divided, letters selected, shapes divided, shapes selected) as standardized mean difference in RT to global targets and local targets as described in ref. 14. Similarly, in the Kimchi Palmer Task, we used the difference in decision times for global choices and local choices as a measure of global advantage^26^, as it has previously proven sensitive to sex differences. The rationale was to obtain a comprehensive measure of global vs. local processing by predicting a latent factor of global advantage from these 5 global advantage effects via confirmatory factor analysis. This latent factor of global advantage would be free of potential moderating influences of stimulus material, attention condition or task and therefore represent the individual processing style irrespective of the assessment situation. However, a confirmatory factor analysis combining all five global advantage effects in one latent global advantage factor did not converge. Subsequent parallel analyses indicated that in fact, each global advantage effect represented an independent factor, i.e. there was no underlying individual style of global-local processing to be captured irrespective of the assessment situation. The corresponding correlation matrix (Table 1) shows that indeed there is no interrelation between the five variables, except for a trend association between the GA in the letters divided and shapes divided condition, which did however not survive a Bonferroni corrected threshold. Therefore, the five GA effects had to be utilized as separate independent variables.

**Table 1:** Correlations between the global advantage effects in the 4 conditions of the Navon task and the Kimchi-Palmer task.

	Letters divided	Letters selected	Shapes divided	Shapes selected
Letters selected	−0.03			
Shapes divided	0.26~	−0.05		
Shapes selected	−0.01	0.14	0.09	
Kimchi	0.04	0.06	0.01	0.04

Throughout the manuscript, linear mixed effects models are implemented using the lmer function of the lme4 package. All models enter participant number as a random factor to control for repeated measurements and are described in detail in the respective paragraphs of the results section. In all models non-significant interactions are backwards eliminated at a Bonferonni-corrected threshold. In a first step, sex differences and menstrual cycle dependent effects on the global advantage effect in the Navon paradigm were addressed using linear mixed effects models with the GA as dependent variable. In a second step, all GA effects were entered as independent variables in linear mixed effects models on measures of cognitive strategy in the cognitive tasks. These measures include the perspective effect and strategy effect in the navigation task, the average cluster size and number of switches in the phonemic and semantic verbal fluency task.

In order to obtain standardized effect sizes, dependent and independent variables were scaled in all analyzes. That way, the estimate b represents an effect size based on standard deviations similar to Cohen’s d.

Data and scripts are openly available at http://webapps.ccns.sbg.ac.at/OpenData/.
